# Round the Hospitals

**Published:** 1918-04-13

**Authors:** 


### ROUND THE HOSPITALS.

The Roll of Honour attached to the Order of the Memorial Service for Nurses who have fallen in the War (see page 31) held at St. Paul's Cathedral on April 10 is a touching and eloquent document which will assuredly survive for the admiration of future generations. In it are enshrined the names of 169 nurses and of 183 members of Voluntary Aid Detachments. Their lives have all been lost in the course of the war, most, it may be inferred, from illnesses incurred in the discharge of their duties, many through the lawless acts of the enemy on sea and on land. Out of the Q.A.I.M.N.S. five nurses and sisters have died. The Q.A.I.M.N.S.R. shows a death roll of sixty-nine, of whom twenty-four were drowned, three were "accidentally killed," and two were "killed," a distinction of enormous significance. Of Q.A.R.N.N.S. one member was drowned; two were drowned and one died of the Reserve. Three members of the Colonial Nursing Association were drowned. The T.F.N.S. has a list of twenty-nine nurses, one of whom was drowned, one died of wounds, and three were killed. Eleven nurses of the Joint War Committee died, including one who was accidentally killed. Twelve nurses attached to the Scottish Women's Hospital lost their lives, one of whom was killed and another died of wounds; this was an exceptional death toll for such a small force to pay. Eight of the Australian Army Nursing Service, and seven of the Canadian A.M.S., two of the Indian M.N.S., fourteen of the New Zealand N.S. (ten of whom were drowned and one accidentally killed); and three of the South African N.S., lost their lives. Four members of the Serbian Relief Fund died, and one nurse in France unattached. Lastly comes the name of Edith Cavell, in Belgium, "executed."

If the tales of heroism untold in this bare record could but be revealed, there would be enough to inspire the women of the Empire for all time. A few such incidents have been collected recording the manner in which these heroic women surrendered their lives, and we would gladly have more. Of Sister Julia Winchester, C.N.A., trained at Hendon Infirmary, for instance, who was drowned on the *Falaba*, we learn "she was a quiet, unassuming woman, always thinking of others." When the torpedo struck the vessel she gave up her life-belt and stood back for someone else to take her place." A lifetime of self-abnegation paved the way for this instinctive act, typical of the profession.

The nomination papers in connection with the election of members of the Council of the College of Nursing are now in the hands of the members, and must be returned by April 17. As a new feature in elections of this kind, the act of nomination requires some care, and its distinction from the act of election may not be at once apparent to all entitled to use the privilege. Many nurses may feel disinclined for the responsibility of nominating members of the Council, but groups of nurses in institutions will probably agree together to propose some candidate, and in that case, if one signs as proposer and another as seconder, the nomination of their candidate will be as effectual as if they filled up dozens of forms. Should any member of the College experience difficulty in securing a seconder for her candidate, it would be worth her while to try whether some present member of the Council would help in this way. The announcement that members may nominate nurses for the Council who are not yet members of the College may cause some surprise. But in every case the nominee must give on the nomination paper her written consent to accept office, which of course implies a desire to join. In the present stage of affairs, when the College is receiving new adherents so rapidly, the authorities believe the time has not come for limiting the scope of choice. The Register of the College consists of an English and Welsh, a Scottish and an Irish section, and of the vacancies, twelve in number, eight will be nominated by the former, and two each by the latter sections. The business of nominating and voting correctly for the College Council is good practice in public affairs, in which women are now to take a more intimate part.

It is significant of the future intentions of the College of Nursing to find Sir Arthur Stanley at Exeter deploring the fact that there is neither recognition of the nurses' uniform nor protection for it. Undoubtedly the Joint Committee has used its brains to some purpose in the matter of ensuring protection for the uniform of its own nurses and helpers, and it may be surmised that when the time comes the means adopted for safeguarding the garb of a trained nurse will be on much the same lines. Unless an absolutely bizarre costume be designed for nurses it would be impossible to prevent nursery-maids and impostors from sailing so near the wind that endless irritating mistakes would arise. There is no form of badge which could not be imitated in such a way as to deceive the public and yet screen the wearer from a breach of the law. But the system of a badge combined with a bar bearing the number of the county, contingent, or whatever form of territorial subdivision might be agreed upon, ensures complete protection both to the nurse and to the public, for reference to headquarters would immediately expose the impostor. In fact the certainty of detection under this system is such that we do not know any instances of women venturing to wear the V.A.D. uniform when they were not entitled to do so.

A most successful meeting of College of Nursing members was held on April 9 in the Lecture Room of the Leicester Royal Infirmary Nurses' Home, for the purpose of considering how best to form a Local Centre of the College in Leicester. Fifty-four members of the College of Nursing were

**ROUND THE HOSPITALS—(*continued*).**

present, and were most enthusiastic on the subject. A local representative, Miss Masters, who will also act as Hon. Secretary, was appointed, as well as a small General Committee. On this Committee general nursing will be represented by Miss Conway Jones, R.R.C., Sister in the Leicester Territorial Hospital; Poor-Law nursing by Miss Masters, Matron of the North Evington Poor-Law Infirmary; Town district nursing by Miss Mearns, Superintendent of the Queen's Nurses in Leicester; County district nursing by Miss Canty, Superintendent of the County District Nursing Association; Private nursing by Miss Disney, Superintendent of the Leicester Trained Private Nurses' Co-operation; Fever nursing by Miss Davies, A.R.R.C., Matron of Gilroes Isolation Hospital, Leicester. Miss Vincent, R.R.C., Matron of Leicester Royal Infirmary, has consented to act as Chairman of the Committee. It was decided that an annual subscription of 2s. 6d., to go towards the working expenses of the local branch, should be contributed by each member. The meeting further decided to arrange for a course of post-graduate lectures in connection with the Leicester Local Centre. The course will begin in October and extend to March, and the cost is to be 5s. for the course, or 1s. per lecture. The lectures will be open to all nurses, whether members of the College or not. The eneregy and progressive spirit displayed at this meeting are worthy of Leicester Royal Infirmary, and those responsible for the formation of the Leicester Centre may indeed feel encouraged to go ahead. An important meeting of the Nation's Tribute Fund for Nurses is arranged for April 25 at the Infirmary, when the Hon. Sir Arthur Stanley will explain the objects of the College. The High Sheriff of Leicester, Mr. T. Fielding Johnson, junr., will be in the chair, and Mrs. Martin Harvey, the Mayor of Leicester, and Colonel C. J. Bond, C.M.G., will speak. Mr. Martin Harvey has consented to recite.

---

The meeting at the Birmingham General Hospital on April 2 of members of the College of Nursing unanimously resolved to form a Local Centre. Miss Bodley, Matron of Selly Oak Infirmary, was elected Local Secretary, and Miss Blantyre Shaw (Walsall) Assistant Secretary. All College members who have not already done so are asked to send their names and addresses to Miss Blantyre Shaw, Lyndhurst, Buchanan Road, Walsall.

---

Many thousands of hospital nurses, and, indeed, district nurses also, throughout the country are affected by the new Franchise Act, which will shortly come into operation. The preliminary steps in connection with the compilation of the new register are being everywhere taken, and forms of inquiry will be sent through the post to householders and occupiers very shortly. The qualifying period of residence or occupation for the purpose of the Act has been fixed to end on April 15. It is an accepted principle that, apart from the more usual franchise, a woman of the requisite age may qualify for the Parliamentary vote as a service voter or as the hirer of unfurnished rooms of the yearly value of £5 or over. Both these qualifications affect a large number of hospital and other nurses, especially, of course, the service qualification or occupation by virtue of employment. The first publication of the voters' lists by the local authorities is fixed for June 15. Nurses who believe they are entitled to figure in the lists but find their names missing should communicate at once with the overseers or the local authority. The lists will be found attached to church and chapel doors, or may be consulted at the public offices of the town.

---

Are newly-certificated nurses joining up from the training-schools in sufficient numbers to keep things going smoothly in France? The Supply of Nurses Committee estimated at the beginning of last year that some 3,600 nurses from Poor-Law infirmaries and civil hospitals in the United Kingdom would complete their training in the course of the following twelve months. They considered that out of this number they ought to get about two-thirds for military nursing, or 2,400. It is highly necessary for the heads of the training-schools to be informed whether this estimate has proved correct. The Committee believed they might reckon upon 1,000 nurses for military purposes from the Dominions, but did not anticipate that more than 300 nurses were likely to volunteer from among the ranks of private nurses attached to civil hospitals, nursing homes, and institutes. Evidently nurses for military service do best when taken straight from their training-schools. It is impossible to over-emphasise the gravity of the present situation and the paramount claim of the nation to the services of every newly-certificated nurse, regardless of future prospects. Last year difficulties about leave in the Expeditionary Force were almost entirely due to a shortage in the numbers available for military service, and now that the same grievance is being pressed with even greater force, the authorities would be wise to tell the nursing profession plainly to what extent nurses have been coming in and how many more they must have. It does not do to leave these things to chance inclination. It is the immediate and pressing duty of all young nurses to go.

---

It seems likely that the Domestic Science School will ultimately solve the problem of how to bridge the gulf between the time when girls leave school and the time when they can begin to receive training as nurses. Dr. Leslie Mackenzie, as we recently pointed out, considered this kind of training an essential feature in the making of the health visitor, and it would be no less useful to nurses preparing for private and institution work. Domestic Science is at present in a very early stage of development, but already it attracts a considerable body of serious students in addition to crowds of mere ordinary girls, who find it distinctly more congenial than book-work. The

**ROUND THE HOSPITALS—(*continued*).**

students are not all of tender age. Many women between twenty and thirty are found putting themselves to school in order to learn home crafts. Hence it is very desirable that matrons who feel the need for promising probationers should put themselves in communication with the heads of such Domestic Science Schools as are found in their locality, and place at their disposal full information as to the advantages and requirements of their training-schools.

---

It is good, in the period of cruel suspense through which the nation is passing, to recall the splendid triumphs wrought by the men and women of our race in the teeth of defeat, disaster, and despair. The Scottish Women's Hospitals in the war zones, now appealing for more funds to carry on their work, are seven in number—two in Salonika, one in Macedonia, one in Corsica, and three in France. They cost £8,000 a month, and Miss Vera Holmes, who joined Dr. Inglis from the outset in her heroic labours in Serbia, has been lecturing on their behalf. She and a few others remained behind with Dr. Inglis to share the retreat of the Serbs and tend them "till none remained who needed their care," and she recalls the crisis which occurred when every conceivable place was filled with the wounded and the enemy were advancing. Dr. Elsie Inglis is related actually to have continued her surgical labours for fifty-nine hours at a stretch, on one occasion at Galatz. The hospital was in a loft and the patients had to be lifted up a ladder to the operating-room. They finished their work before the enemy arrived, and all the patients were safely evacuated. To have shared the honours of such deeds was worth living for, and twelve nurses, whose names occur in the Roll of Honour, have died to share it.

---

A painful sensation has been created among Melbourne nurses by the sudden death of a nurse, in her fourth year, at the Melbourne Hospital. It appears that she went on duty after recovery from a somewhat severe illness and, unhappily, without the usual examination by the medical superintendent. She broke down the same day, and died nine days later. It can hardly be imagined that the fatal end was brought about by the few hours' return to work, but, as often happens, the case has crystallised a good deal of dissatisfaction about nursing conditions in general. Certain people are pressing for the formation of a Nurses' Union designed to bring about the reduction of the nurses' training from four to three years, the abolition of scrubbing and "heavy work" for the probationer, the establishment of "an eight hours' shift with an hour for meals in each shift," and the attainment of a "good living wage," "daily motor-rides to obtain fresh air," and other *desiderata*. They also want to have what they call "the nurse's doom" vested in a board of appeal, consisting of the matron and two others, instead of leaving it to be decided by the matron alone. It is a common experience that unions aiming at purely material alleviations and uninspired by ideals seldom effect much, and Melbourne nurses must improve their outlook and study the higher needs of the profession if they want to be taken seriously.

---

The South African Medical Bill has been dropped, and with it the hopes of South African nurses of obtaining the compulsory registration of trained nurses practising their art, with the establishment of penalties against unregistered women practising for gain. In each of the four South African provinces there is a Medical Council, which has control of the nurses in its own province, examines probationers, registers them on completing their course, and is responsible for the status and welfare of the nursing profession. It also registers nurses coming in from outside. But the registration is voluntary, and there is nothing to stop an unregistered woman from practising as a nurse or as a midwife. The position of the coloured nurse is still a great problem. The Transvaal Medical Council declares it has no power at present to limit the training of any class or to institute a second-grade examination and certificate. So coloured women, who for the most part are quite incapable of passing nursing examinations, practise as nurses after employment at little hospitals, and are under no supervision. That their practice should be controlled and their work confined within well-defined limits are precautions which cannot long be neglected on grounds of public safety.

---

The increased cost of domestic help presses very hardly on many hospitals in this country. But the domestic difficulty has not yet been accentuated to quite the same extent as in America. Our contemporary, *The Trained Nurse*, has been asking hospital managers to what extent the wages of such employees have increased since 1914, and has received answers which testify to a rise of from 25 to 50 per cent. in wages, while in most cases the quality of the service is stated to be "exasperatingly poor" as compared with pre-war conditions. One manager writes: "Our floor help is now taken care of by coloured men, whom we pay $2.75 per day and three full meals. This same work was formerly taken care of by a Polish maid at $20 per month and one meal." The attractions of the munition factory appear to be largely responsible there, as here, for the shortage of maids. But the fact is that modern progress demands the extinction of cheap untrained labour, and the only real solution of the domestic problem lies in making the kitchen- and ward-maid service a training-school, with good hours, good wages, and good chances of promotion.

---

**☛ It is our invariable practice to pay for all items of news which we may publish. The minimum payment is 5s. Paragraphs of about twenty lines in length—that is to say, of 150 words or less—are preferred. Contributions should be addressed to The Editor, The Hospital, 28 and 29 Southampton Street, Strand, London, W.C. 2, and, to be in time for the current week's issue, should reach him by the first post on Mondays.**

For St. Paul's Cathedral Memorial Service to Nurses, see p. 31; Nursing Affairs in Ireland, p. 32.

